# Reactivity of Gold(I) Monocarbene Complexes with Protein Targets: A Theoretical Study

**DOI:** 10.3390/ijms20040820

**Published:** 2019-02-14

**Authors:** Iogann Tolbatov, Cecilia Coletti, Alessandro Marrone, Nazzareno Re

**Affiliations:** Dipartimento di Farmacia, Università degli Studi “G. D’Annunzio” Chieti-Pescara, Via dei Vestini, I-66100 Chieti, Italy; tolbatov.i@gmail.com (I.T.); cecilia.coletti@unich.it (C.C.); alessandro.marrone@unich.it (A.M.)

**Keywords:** anticancer, N-heterocyclic carbenes, gold(I) complexes, DFT calculations, proteins

## Abstract

Neutral N–heterocyclic carbene gold(I) compounds such as IMeAuCl are widely used both in homogeneous catalysis and, more recently, in medicinal chemistry as promising antitumor agents. In order to shed light on their reactivity with protein side chains, we have carried out density functional theory (DFT) calculations on the thermodynamics and kinetics of their reactions with water and various nucleophiles as a model of plausible protein binding sites such as arginine, aspartic acid, asparagine, cysteine, glutamic acid, glutamine, histidine, lysine, methionine, selenocysteine, and the N-terminal group. In agreement with recent experimental data, our results suggest that IMeAuCl easily interacts with all considered biological targets before being hydrated—unless sterically prevented—and allows the establishment of an order of thermodynamic stability and of kinetic reactivity for its binding to protein residues.

## 1. Introduction

The administration of gold complexes has been assessed for various pharmaceutical aims, particularly in the treatment of cancer and arthritis [1,2,3,4,5,6,7]. A plethora of gold(I) and gold(III) complexes with different ligands display substantial cell-growth-inhibiting properties [4,8]. Nevertheless, taking into account the great structural variation of their ligands, it seems that there is no common mode of action that could describe all of them [4,6,8]. Gold compounds target various cellular processes that ultimately activate apoptosis: direct DNA damage, mitochondrial damage via thioredoxin reductase inhibition, proteasome suppression, the modification of the cell cycle, and the modulation of specific kinases [1,2,3,4,5,6,9,10,11,12].

Gold(I) N–heterocyclic carbenes (NHC) of the general formula [Au(I)(NHC)R], in which R is an anionic or a neutral ligand such as Cl^−^, PH_3_, or another NHC moiety, leading to a neutral or to a cationic complex, have been extensively utilized in homogeneous catalysis [13,14,15,16] and, more recently, in medicinal chemistry [4,17,18]. Their unique chemical and biological properties make them successful anti-arthritic [19], antibacterial [20], and anticancer [18,21,22,23,24,25,26,27] agents. At variance with platinum-based drugs, DNA is not the main pharmaceutical target for these gold-based cytotoxic agents that, conversely, mainly act through the modification of selected proteins with consequent loss of function. This implies that selective ‘‘protein metalation’’ is the key feature in the mechanism of action of anticancer gold drugs. In particular, several studies have recently highlighted that the crucial role in the mode of action of N–heterocyclic carbene gold(I) complexes emerges from an efficient selective inhibition of the enzyme thioredoxin reductase (TrxR) [1,7,25,28,29]. Electrospray ionization mass spectrometry (ESI-MS) measurements indicate that gold carbene complexes preferentially attach to free cysteine and selenocysteine side chains [19,30], with the thiol/selenol group being coordinated by [Au(NHC)]^+^ fragments or Au^+^ ions upon the release of carbene ligands. Linear Au(I) complexes have also been shown to be effective inhibitors of the Se-free enzyme glutathione reductase (GR), owing to the high thiol reactivity toward gold, as plainly manifested by the crystal structure of GR inhibited by a gold(I) complex displaying Au(I) attached to the active site of Cys thiol with linear S–Au–S coordination [23,27,31]. Detailed ^1^H NMR studies of the ligand exchange reactions of cationic Au(I)(NHC) complexes with cysteine and selenocysteine reveal a two-step reaction comprising the consecutive substitution of both NHC ligands to yield the [Au(Cys)_2_] compound [25]. Although as a soft metal ion Au(I) shows a marked preference for soft ligands such as thiols of cysteines and thioethers of methionines [32], multiple X-ray crystallography data have shown that Au(I) ions can coordinate solvent-exposed His [33,34,35], even in the presence of free thiols. In particular, a recent X-ray study [36] has shown that in the absence of free cysteines, methionines, and histidines, these Au(I)(NHC) gold compounds are able to bind the side chains of Lys and Arg residues or the N-terminal Ala amino group. 

**Scheme 1:**
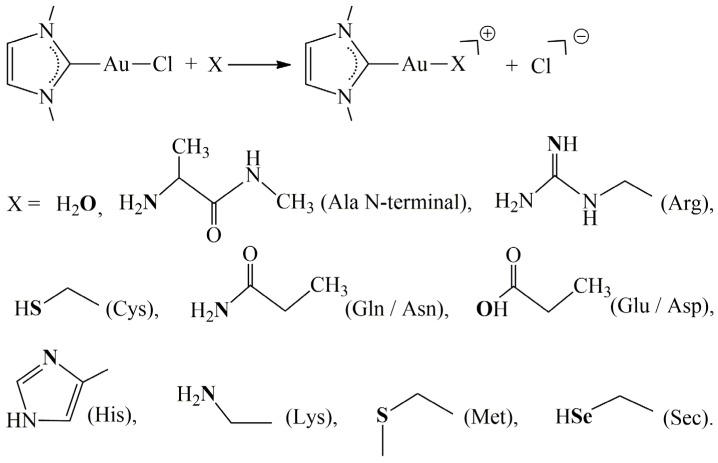
Reaction scheme and considered nucleophiles. Attacking atoms in **bold**.

Even though the interaction of gold carbene compounds with many proteins (for example, TrxR, phosphatases, glutathione reductases, serum albumin, Atox-1, or thaumatin) has now been widely examined [1,7,17,19,25,28,29,30,36,37,38,39,40,41,42,43,44], their detailed mechanisms of reaction with protein side chains are still lacking. In this study, we compute the reactivity of the neutral Au(I)NHC complex (Me_2_Imy)AuCl, or IMeAuCl, with water, and with the main binding sites in a protein or a polypeptide, i.e., the side chains of arginine, aspartic acid, asparagine, cysteine, glutamic acid, glutamine, histidine, lysine, methionine, and selenocysteine residue, and the N-terminal group. We have used short models of these residues (Scheme 1). Each side chain was represented by the nucleophilic group, while the rest of the chain was represented by the ethyl group. A deeper insight into the binding mode of these Au(I) complexes with protein targets and their binding preference would be very useful to fully comprehend their mechanisms of action in vivo and may be useful to conceive novel and more effective gold(I) anticancer drugs.

## 2. Results and Discussion

**Figure 1:**
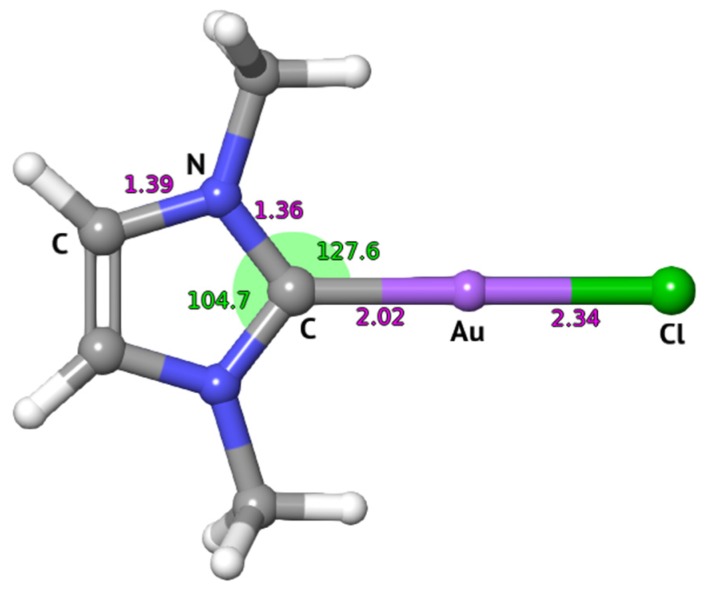
Calculated geometrical structure of the IMeAuCl complex. Bond lengths (purple in the figure) in Å, angles (green in the figure) in degrees.

A preliminary investigation on the isolated IMeAuCl complex has shown that at the selected level of theory, the minimized structure matches the crystallographic data [45] accurately with Au–C and Au–Cl bond distances within 0.05 Å, and Cl–Au–C angles within 2°, thus indicating that the B3LYP functional provides a good description of this molecular structure (Figure 1, Table S1). 

We first considered a dissociative mechanism with detachment of a chloride anion. The reaction barrier of 26.5 kcal/mol and reaction energy of 13.1 kcal/mol make the dissociation unlikely. DFT calculations were therefore carried out on the thermodynamics and the kinetics of the ligand exchange of one chloride ligand by incoming nucleophile molecules. Simplified models of the residue’s side chains (Scheme 1) were preferred to either free or capped amino acids for several reasons. First, free amino acids are less representative of protein residues due to the presence of terminal amine and carboxylic acid groups, in ionized zwitterionic form, which do not exist in proteins. The capped forms, although chemically resembling protein residues, are connected to the side chain nucleophilic group through hydrocarbon chains of different lengths, thus leading to large variations in the size of the ligand reacting with the metal complex. This latter aspect may bias the calculation of solvation free energies, thus affecting the comparison of amino acid reactivity. All residues were assumed to be in their most stable protonation state at pH=7.2, i.e., positive for arginine, lysine, and N-terminal alanine; neutral for histidine, cysteine, methionine, and asparagine/glutamine; and anionic for selenocysteine and glutamic/aspartic acid. However, for arginine, lysine, and N-terminal alanine, we considered as nucleophiles the neutral form taking into account the deprotonation energy. For cysteine, we considered both the neutral form, expected to be the most stable at neutral pH and the anionic form, which is still present in low concentrations, or is even the most stable form, when cysteine is close to basic residues (such as histidine) which are known to stabilize the anionic form. 

The reaction was investigated, taking into account that both reagents and products can form stable non-covalent adducts both prior to and after the reaction takes place. We have thus optimized the geometries of the reactant adducts (RA), transition states (TS), product adducts (PA) intermediates, and their energies with respect to the isolated species that have been evaluated and given below. The activation enthalpies and free energies have been calculated as the difference between TS and the lowest between reactants and reactant adducts, while the reaction enthalpies and free energies have been calculated as difference between reagents and products infinitely apart. The calculated values for the IMeAuCl reactions with all considered molecular models are reported in Table 1, and the results allow one to establish an order of thermodynamic stability and kinetic reactivity for the Au(I) binding to the considered protein residues side chains. 

The results were compared to recent experimental X-ray and ESI-MS studies of the binding of this Au(I)NHC neutral complex to peptides and proteins [30,46] showing Au(I) binding mainly to cysteine and selenocysteine, and to the more recent results of the paper by Merlino et al. [36] reporting the first distinct crystal structure of a gold N–heterocyclic carbene compound bound to a protein—thaumatin—showing the Au(I) metal center binding lysine, arginine side chains, as well as the N-terminal group. 

**Figure 2:**
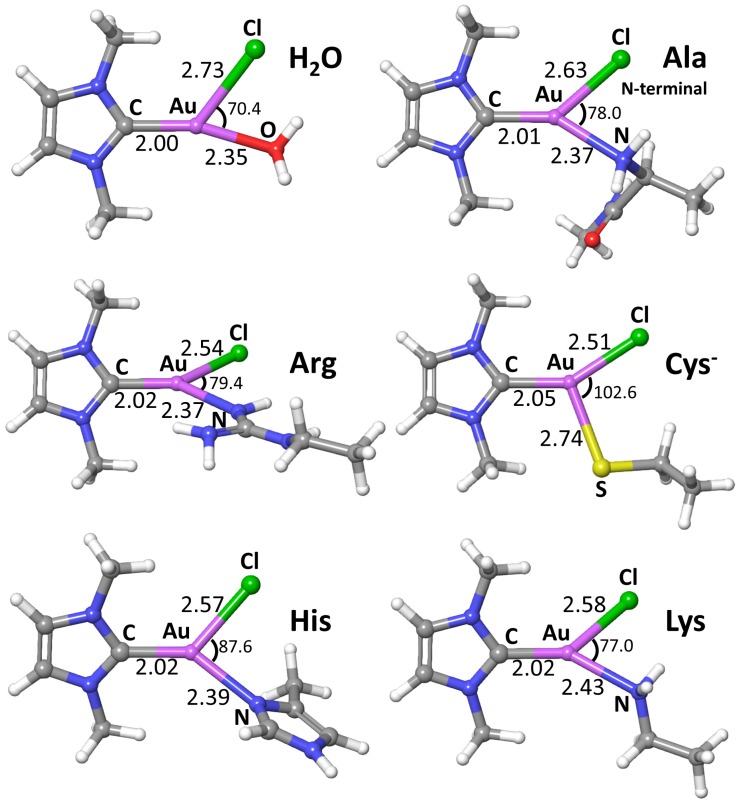
Transition states for selected nucleophiles. All distances in angstroms, all angles in degrees.

We first considered the hydrolysis reaction with substitution of the chlorido ligand by a water molecule to probe the stability of this chloro NHC complex in biological fluids. The transition state (imaginary frequency of −62.92 cm^−1^) (Figure 2 and Figure S2) for the reaction with water is characterized by the entering water molecule approaching the metal center with Au–O distance of 2.35 Å, whereas the leaving chlorine moves further at the distance of 2.73 Å. 

**Figure 3:**
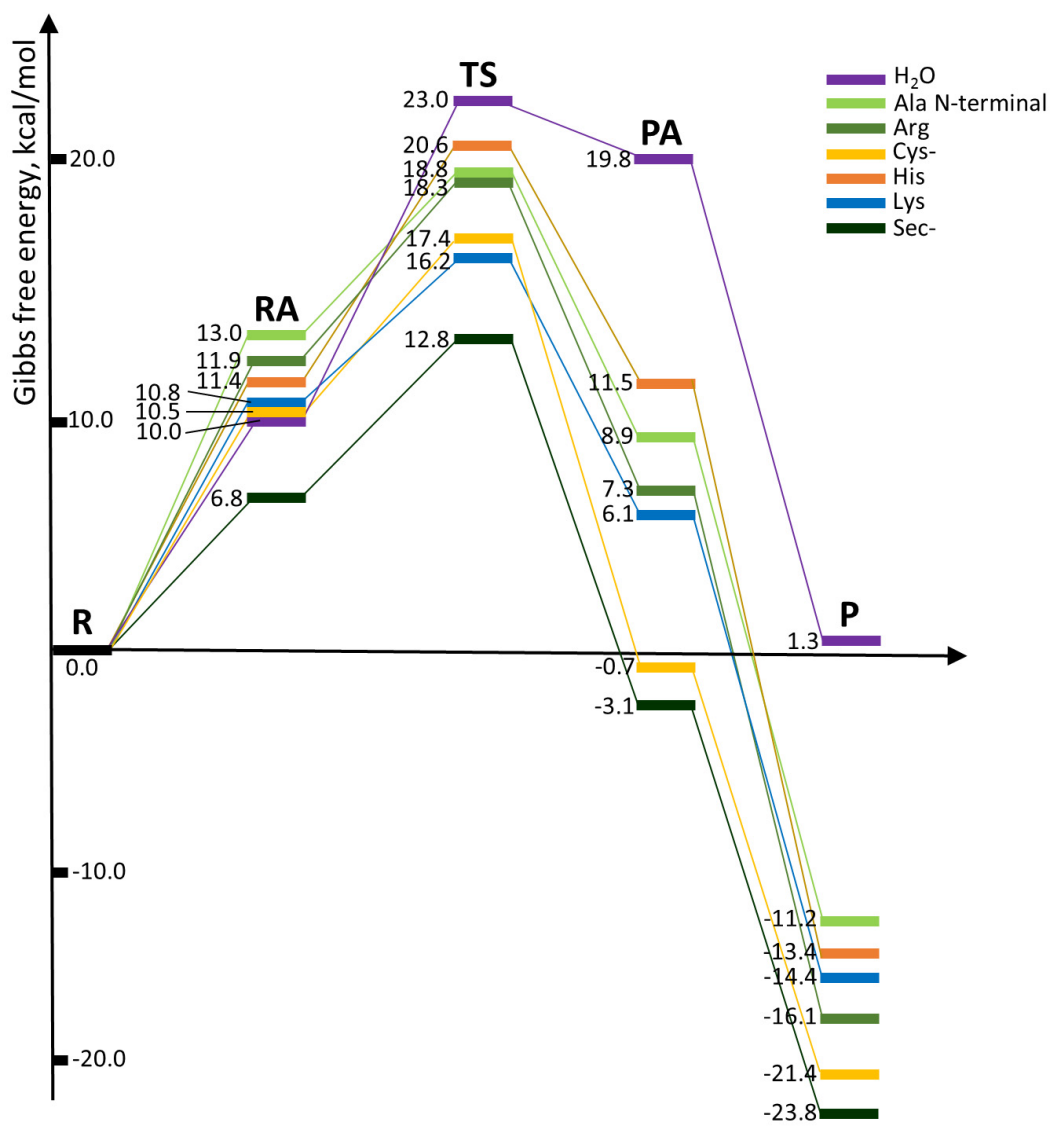
Reaction profiles for IMeAuCl interacting with selected nucleophiles. Values in kcal/mol are calculated at the B3LYP/LACV3P**++//B3LYP/LACVP** level. R, P, TS, RA, and PA stand for reactant, product, transition state, reactant-adduct, and product-adduct, respectively.

An approximately planar trigonal geometry is observed, with an acute leaving ligand—metal—entering ligand angle of 70.4°. Table 1 and Figure 3 and Figure S3 show that this reaction is slightly endergonic, with a reaction-free energy of 1.3 kcal/mol. 

The calculated activation-free energy of 23.0 kcal/mol predicts this reaction to be relatively fast at the physiological temperature, although its barrier is significantly higher than that of all other nucleophiles examined in the present study, indicating that the IMeAuCl complexes are likely to attack the biological targets before being hydrated. Although there are no experimental data for the barrier of the hydrolysis of any [Au(I)(NHC)Cl] complex, nor is there any clear indication of its stability in solution or neutral buffer, the calculated value of the activation-free energy is reasonably consistent with the sparse experimental evidence showing that chlorido 1-butyl-3-methyl-imidazol-2-ylidene gold(I) complex is stable for at least 24 hours at room temperature [47], while chlorido 1,3-methyl-benzimidazol-2-ylidene gold(I) complex undergoes hydrolysis processes within a few hours in buffer solution pH 7.0 incubated at 37 °C [48]. The B3LYP results for this prototypical process were benchmarked against latest-generation density functionals, such as M06-2X, wB97X, and CAM-B3LYP-D3, which have been recently shown to be among the best-performing density functionals in the calculations on main group molecules and metal complexes [49,50,51] and against more accurate ab initio calculations such as MP2 with larger basis sets. The results are reported in Table S2 and show that reaction and activation enthalpies and free energies within 2–3 kcal/mol form the B3LYP results, thus indicating that the B3LYP functional provides a good description of the energetics of this kind of process.

Transition states for methionine, cysteine, and selenocysteine in their neutral form (with imaginary frequencies of −50.43, −44.33, and −18.09 cm^−1^, respectively) have similar planar trigonal geometries, with gold–sulfur/selenium and gold–chlorine distances being 2.6–2.7 A and the S/Se–Au–Cl angle varying within 82–88°, and show analogous reaction profiles with reaction activation barriers of 18.2, 18.1, and 17.6 kcal/mol, respectively. When cysteine and selenocysteine are considered in their anionic form, lower activation energies have been calculated, respectively, 15.5 and 12.8 kcal/mol, as expected on the basis of the better nucleophilicity provided by the negative charge. 

**Scheme 2:**
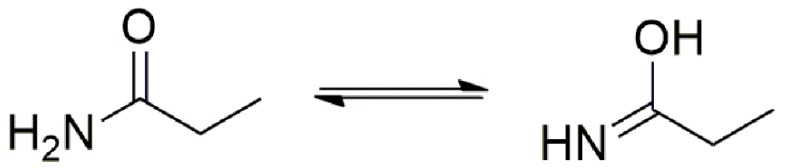
Amidic/imidic tautomer equilibrium of glutamine/asparagine.

Nucleophiles attacking with their N-based moieties, His, Arg, Lys, and N-terminal amine, form planar trigonal transition states with slightly smaller angles within 77–79°, and Au–N distances of 2.3–2.4 Å, with imaginary frequencies of −59.6 −61.8, −60.8, and −52.9 cm^−1^ (Figure 2 and Figure S2). Reaction activation barriers for the N-based moieties are also quite low in this case: 20.6 kcal/mol for histidine, 18.3 kcal/mol for arginine, 16.2 kcal/mol for Lys, and 18.8 kcal/mol for N-terminal. When binding to glutamine/asparagine is considered, two different tautomers could in principle be taken into account, corresponding to the amide and imide forms (Scheme 2): for both tautomers, a TS was found with geometries similar to those of the other N-based nucleophiles and activation-free energies of, respectively, 24.8 and 16.8 kcal/mol. 

The barrier for the Cl substitution by the imidic form is much lower than that of the amidic form, but it should be born in mind that the imidic form is significantly less stable, by 16.4 kcal/mol, and is stabilized only in few protein systems by suitable adjacent catalytic residues [52] (Figure S1).

Finally, binding to glutamic/aspartic acid was considered, and we found a TS with the usual distorted trigonal geometry and a relatively high activation free energies of 24.7 kcal/mol.

The results for chloride substitution from the other residue models are reported in Table 1 and show that all reactions are slightly to moderately exergonic with the following order of thermodynamic stability:

Sec > activated-Cys > Arg > Lys > His > activated-Gln/Asn > N-terminal > Met > Cys > Glu/Asp > Gln/Asn >

although the kinetic reactivity order is quite different:

Sec > Lys > activated-Gln/Asn > activated-Cys > Cys > Met > Arg > N-terminal > His > Gln/Asn ≈ Glu/Asp >

These results are consistent with the experimental evidence showing that the selenocysteine residue of thioredoxine, and, to a lesser extent, the histidine-activated cysteine residues of several enzymes such as glutathione reductase, react promptly with neutral Au(I)(NHC) complexes and are considered the main physiological targets of this class of compounds. In particular, the low barriers for Cys and especially Sec are in agreement with the results of a recent study on the thioredoxin reductase inhibition by [Au(NHC)Cl] and on the reaction of the same complex with the reduced linear dodecapeptide Ac-SGGDILQSGCUGNH_2_ investigated by ESI-MS, showing (i) a relevant inhibition of thioredoxin reductase activity soon after mixing (about 30 s) and (ii) the prevalent binding of a [Au(I)(NHC)]^+^ fragment to the selenocysteine residue and, to a much lesser extent, to cysteine [30].

On the other hand, glutamic/aspartic acid and glutamine/asparagine show high barriers around 25 kcal/mol, and slightly exergonic or even endergonic reaction free energies in agreement with the experimental evidence, indicating that such residues are not good binding sites for [Au(I)(NHC)]^+^ complexes. However, the interaction of some Au(III) complexes with hen egg white lysozyme has been observed to lead, upon metal reduction and ligand loss, to the binding of an [Au(H_2_O)]^+^ gold(I) fragment to a glutamine residue, probably preceded by a amide-imidic acid tautomerization of the glutamine [46]. 

Binding to histidine shows the highest barrier after glutamic/aspartic acid and glutamine/asparagine, 20.6 kcal/mol, and is therefore slower than the substitution by all other residues, although the barrier is low enough to make the reaction still relatively fast at physiological temperature. However, [IMeAu]^+^ binding to histidine is one of the most exergonic processes involving the neutral residues, so it can be considered to be plausible when metal binding to protein is under thermodynamic control. This result is consistent with previous X-ray studies showing that the reaction of an analogous gold(I) complex—[Au(PEt_3_)Cl]—with cyclophilin-3 forms an [AuPEt_3_]^+^ adduct with an histidine residue, despite the presence of four Cys thiol groups in the protein [53], and that the interaction of several Au(III) complexes with hen egg white lysozyme preferentially leads, upon metal reduction and ligand loss, to the binding of a AuCl gold(I) fragment to histidine, even though several methionine groups are available in the protein [34].

Relatively low barriers are calculated for the N-based nucleophile sites—lysine, arginine, and N-terminal amine (16–19 kcal/mol)—indicating little discrimination in terms of reactivity within either S/Se or N-based nucleophile sites and suggesting that these N-based sites could also bind an [Au(NHC)]^+^ moiety whenever cysteine or selenocysteine residues are absent or buried, and that the binding preference to a protein side chain could be determined by its accessibility rather than by its kinetic preference toward chloride substitution. These results are in agreement with a recent crystallographic study showing that the reaction of 1-butyl-3-methyl-imidazol-2-ylidene gold(I) complex with the model protein thaumatin leads to the binding of the [Au(NHC)]^+^ fragment to lysine and arginine side chains and to the N-terminal amine [36] and suggest that the accessibility of amino acids within the protein rather than reactivity could be the key point to comprehend the amino acid targeting of the [Au(NHC)Cl] complex displayed in the experiment [36]. 

**Figure 4:**
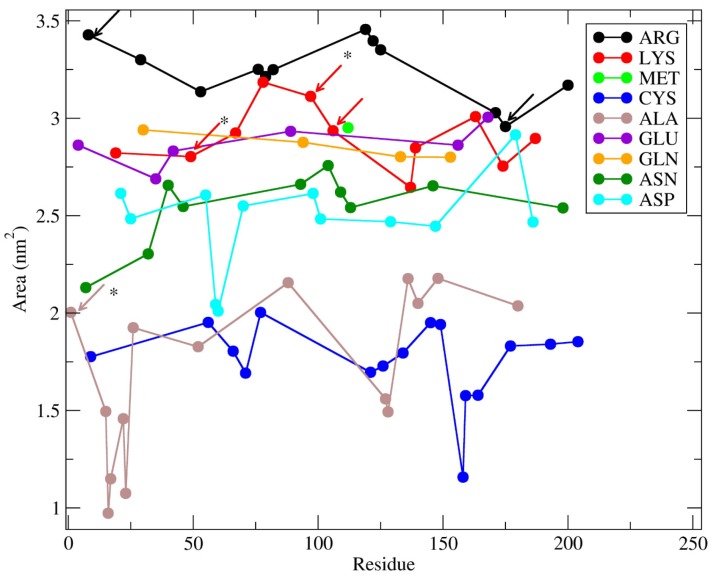
Solvent accessible surface (SAS) calculated on an X-ray structure of Thaumatin-1 (pdb entry 3qy5). Arrows indicate metal bound amino acids. Stars indicate metal centers with an attached carbene ligand.

To better investigate the latter point, solvent-accessible surface (SAS) analyses (Figure 4 caption) were carried out on the X-ray structure of the plant protein thaumatin-1 (pdb id 3qy5) showing that Lys, Arg, Glu, Asp, Gln, Asn, and the only Met residues in thaumatin are markedly more exposed than Cys and N-term, while His residues are absent. 

As shown in Figure 4, the gold(I) centers are always bound to highly-exposed amino acids—mainly Arg and Lys—with the exception of N-terminal amine, which undergoes metal binding despite being less exposed. However, the binding of the [IMeAu]^+^ fragment to the N-terminal amine [36] could be related to the well-known flexibility of the terminal segment of the protein, whose SAS values may vary within a large range in solution despite being restricted to smaller values by the crystal packing. Notably, the preferential Au-binding on Lys106 compared to the sequentially close and similarly exposed Met112 is consistent with the lower values of either reaction or activation-free energies estimated for Lys.

## 3. Materials and Methods 

All calculations were performed with the Jaguar 9.1 [54,55] and Gaussian 09 A.02 [56] quantum chemistry package. Optimizations were carried out in gas phase using the density functional theory (DFT) with the B3LYP hybrid functional [57,58], which is known to give a good description of geometries and reaction profiles for transition metal-containing compounds [59], and particularly antitumor compounds based on metals in different oxidation states [60,61,62,63,64,65,66,67,68] including gold(I) [28,69,70] and gold(III)-based [71,72] anticancer compounds. Other latest-generation functionals explicitly developed for systems with long-range electron correlations were used for benchmarking such as M06-2X [73], wB97X [74], and CAM-B3LYP-D3 [75], which have been recently shown to be among the best-performing density functionals in the calculations on main group molecules and metal complexes [49,50,51], as well as explicitly correlated wave function based methods, such as the Møller–Plesset perturbative approach MP2 [76,77,78].

All geometrical optimizations and single-point electronic energy calculations have been carried out with the LACVP** and LACV3P**++ basis sets, respectively [79,80,81,82]. These basis sets describe the 1s–4d core electrons of the gold atom with the Hay and Wadt core-valence relativistic (i.e., with an implicit treatment of scalar-relativistic effects) effective core-potential (ECP) leaving the outer electrons of gold as well as electrons of the remaining atoms to be treated explicitly by the 6-31G** and 6-311++G** basis sets of double- and triple-ξ quality, correspondingly. Additionally, the basis set aug–cc–pVTZ [83] was used for the MP2 single point energy calculations in order to assess the high-level ab initio methodology performance with a large basis set.

Frequency calculations were performed to verify the correct nature of the stationary points and to estimate zero-point energy (ZPE) and thermal corrections to thermodynamic properties. Intrinsic reaction coordinate (IRC) calculations were employed to locate reagents and products minima connected with the transition states for each considered reaction step. 

Single-point electronic energy calculations were performed on the gas-phase geometries. The Poisson–Boltzmann (PB) continuum solvent method was used to describe the solvation. It represents the solvent as a layer of charges at the molecular surface, serving as a dielectric continuum boundary, thus accounting for the detailed molecular shape [84,85]. Solvation energies were calculated on gas-phase stationary points at the B3LYP/LACV3P**++ level of theory, taking a dielectric constant of 80.37 for water and the standard set of optimized radii in Jaguar. Experimental values have been taken for the solvation-free energies of small ions. Thermodynamic properties in solution were calculated as follows. Solvation-free energies were taken as the difference between the solution energies and the gas phase energies. The calculation of entropic contributions in solution is a rather delicate issue, and is particularly important when entropies (and free energies) need to be evaluated for bimolecular reactions involving separated reactants and products. Indeed, in this case, the translational degree of freedom in the reactants/products becomes a loose vibration for adducts or transition states, leading to a loss/gain of entropy, much larger in the gas phase than in the confined condensed phase. For this reason, the use of gas phase entropies in solution often leads to artificially too large results. A way to overcome this problem was proposed by Werz [86] and proved to lead to solution entropies and free energies in excellent agreement with experimental values [87,88,89,90,91]. Following this approach, a solute dissolved in a solvent loses a constant fraction of its entropy in vacuo. The amount of this loss, for water, can be approximated as 50% of the gas phase entropy changed of sign.

Enthalpies and Gibbs free energies were obtained at 298.15 K and 1 atm from total electronic energies including ZPE, thermal, and entropic corrections, and the addition of solvent corrections. A further correction of ΔG°^→^*=RT ln (24.6) was included to pass from ideal gas at 1 atm to ideal solution at 1 mol/L as standard state [92].

The solvent-accessible surface (SAS) of each considered amino acid was computed by using the *sas* option implemented in Gromacs software [93]. 

## 4. Conclusions

Detailed mechanisms of the reaction of gold carbene compounds with most amino acids and protein fragments remain largely undisclosed. In this study, we have computed the reactivity of IMeAuCl with the various nucleophiles water, side chains of arginine, cysteine, histidine, lysine, methionine, and selenocysteine, and the N-terminal of any amino acid. 

The activation-free energy of 23.0 kcal/mol calculated for the chloride substitution by water makes this reaction relatively fast at a physiological temperature, although it shows a barrier significantly higher than all the other nucleophiles examined in the present study, indicating that the [Au(I)(NHC)Cl] complexes are likely to attack the biological targets before being hydrated. 

The results for chloride substitution from the considered residues show that all reactions are slightly to moderately exergonic with the following order of thermodynamic stability:

Sec > activated-Cys > Arg > Lys > His > activated-Gln/Asn > N-terminal > Met > Cys > Glu/Asp > Gln/Asn >

but have a different kinetic reactivity order:

Sec > Lys > activated-Gln/Asn > activated- Cys > Cys > Met > Arg > N-terminal > His > Gln/Asn ≈ Glu/Asp >

The lowest barriers calculated for Cys and especially Sec are consistent with the experimental evidence showing the prevalent binding of a [IMeAu]^+^ fragment to the selenocysteine residue and, to a much lesser extent, the cysteine residue of thioredoxine.

Relatively low barriers are calculated also for the N-based nucleophile sites, lysine, arginine, and N-terminal amine (16–19 kcal/mol), indicating little discrimination in terms of reactivity within either S/Se or N-based nucleophile sites and suggesting that that these N-based sites could also bind an [IMeAu]^+^ moiety whenever cysteine or selenocysteine residues are absent or buried and that the binding preference to a protein side chain could be determined by its accessibility rather than by its kinetic preference toward chloride substitution.

## Figures and Tables

**Table 1:** Enthalpies/Gibbs free energies for reactions with various nucleophiles. Reported values are calculated at the B3LYP/LACV3P**++//B3LYP/LACVP** level and reported in kcal/mol in format Enthalpy (**ΔH)**/Gibbs free energy (**ΔG)** (in solution). R, P, TS, RA, and PA stand for reactant, product, transition state, reactant-adduct, and product-adduct, respectively.

Reaction with	R → P		R → TS	
	ΔH	ΔG	ΔH	ΔG
H_2_O	7.2	1.3	16.8	23.0
Ala N-terminal	−6.6	−11.2	11.9	18.8
Arg	−11.0	−16.1	11.3	18.3
Cys	−3.5	−9.1	10.9	18.1
Cys–	−16.8	−21.4	11.6	17.4
His	−8.3	−13.4	14.1	20.6
Lys	−10.2	−14.4	9.0	16.2
Met	−5.4	−9.8	11.5	18.2
Sec	−2.2	−6.7	11.2	17.6
Sec–	−19.2	−23.8	6.7	12.8
Gln/Asn (amidic form)	6.0	2.4	17.5	24.8
Gln/Asn (imidic form) *	−7.3	−12.6	10.0	16.8
Glu/Asp acid	−2.2	−7.0	18.2	24.7

* Reactant for the imidic form of Gln/Asn is 16.4 kcal/mol higher than its amidic form, so all the values for the imidic form should be augmented by this value before its comparison with the amidic form. Additionally, see Figure S1.

## Supplementary Materials

Supplementary materials can be found at http://www.mdpi.com/1422-0067/20/4/820/s1.

## Abbreviations

DFT	Density Functional Theory
PB	Poisson–Boltzmann
MP2	Møller–Plesset 2nd order perturbation theory
NHC	N-Heterocyclic Carbenes
