# Supplementary material for: Reactivity of Gold(I) Monocarbene Complexes with Protein Targets: A Theoretical Study
Source: Int J Mol Sci. 2019 Feb 14;20(4):820. doi: 10.3390/ijms20040820 (PMC6412330; doi:10.3390/ijms20040820)
Supplement: Supplementary file 1 [file ijms-20-00820-s001.pdf]

International Journal of
Molecular Sciences

Table S1. Comparison of experimental and calculated values for structural parameters of IMeAuCl complex.

?	Experimental*	Calculated
Au–Cl (Å)	2.288 (3)	2.34
Au–C (Å)	1.979 (11)	2.02
C–N	1.35 (2)	1.36
N–C	1.36 (2)	1.39
C–Au–Cl (º)	178.8 (2)	180
N–C–N	105.8 (10)	104.7
N–Au–C	126.2 (5)	127.6

Table S2. Assessment of various levels of theory for the reaction of a water molecule with IMeAuCl. Values reported in kcal/mol.

Methods	R->P		R->TS	
	ΔH	ΔG	ΔH	ΔG
B3LYP / LACV3P**++	7.2	1.3	16.8	23.0
CAM-B3LYP / LACV3P**++	4.2	-1.7	13.3	19.5
M06-2X / LACV3P**++	2.6	-3.3	8.2	14.5
wB97X / LACV3P**++	4.5	-1.4	11.2	17.4
MP2 / LACV3P**++	10.1	4.2	15.0	21.2
MP2 / aug-cc-pVTZ	9.9	4.0	14.2	20.4

vibrational corrections and solvation energies always calculated with B3LYP/LACVP** and B3LYP/LACV3P**++, respectively

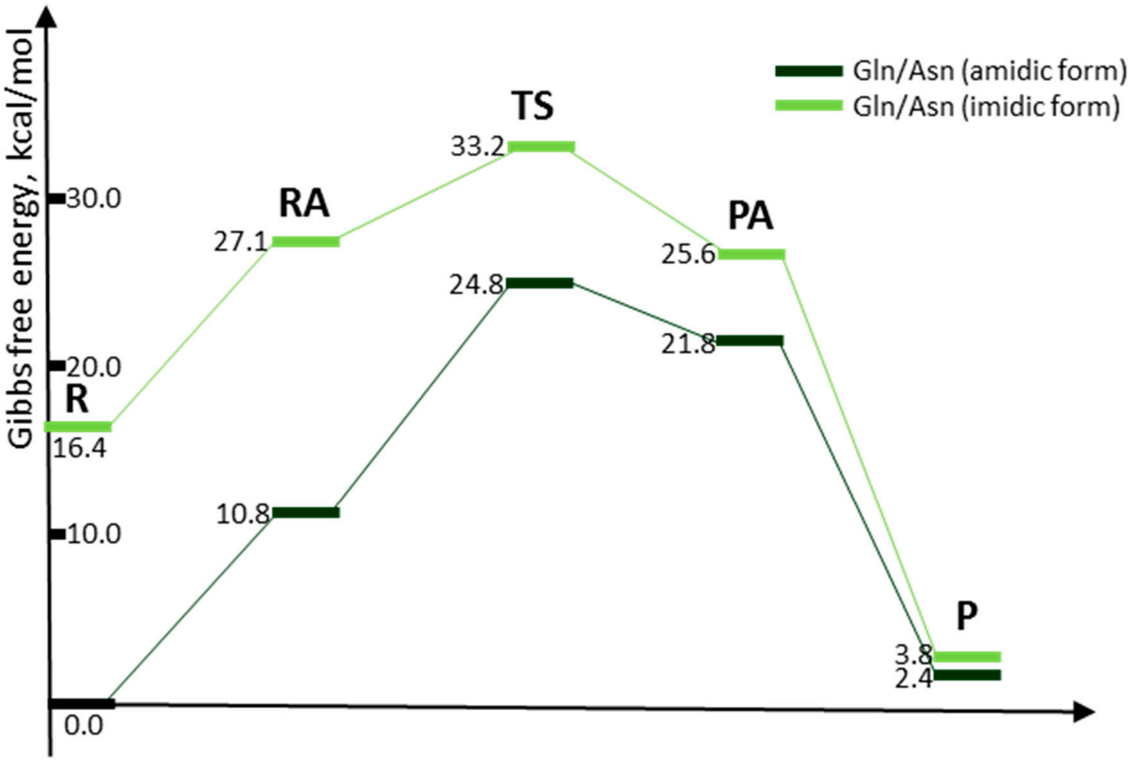

Figure S1. Reaction profiles for the Cl substitution by the amidic and imidic forms of glutamine / asparagine. Values in kcal/mol are calculated at the B3LYP/LACV3P**++//B3LYP/LACVP** level. R, P, TS, RA, and PA stand for reactant, product, transition state, reactant-adduct, and product-adduct, respectively.

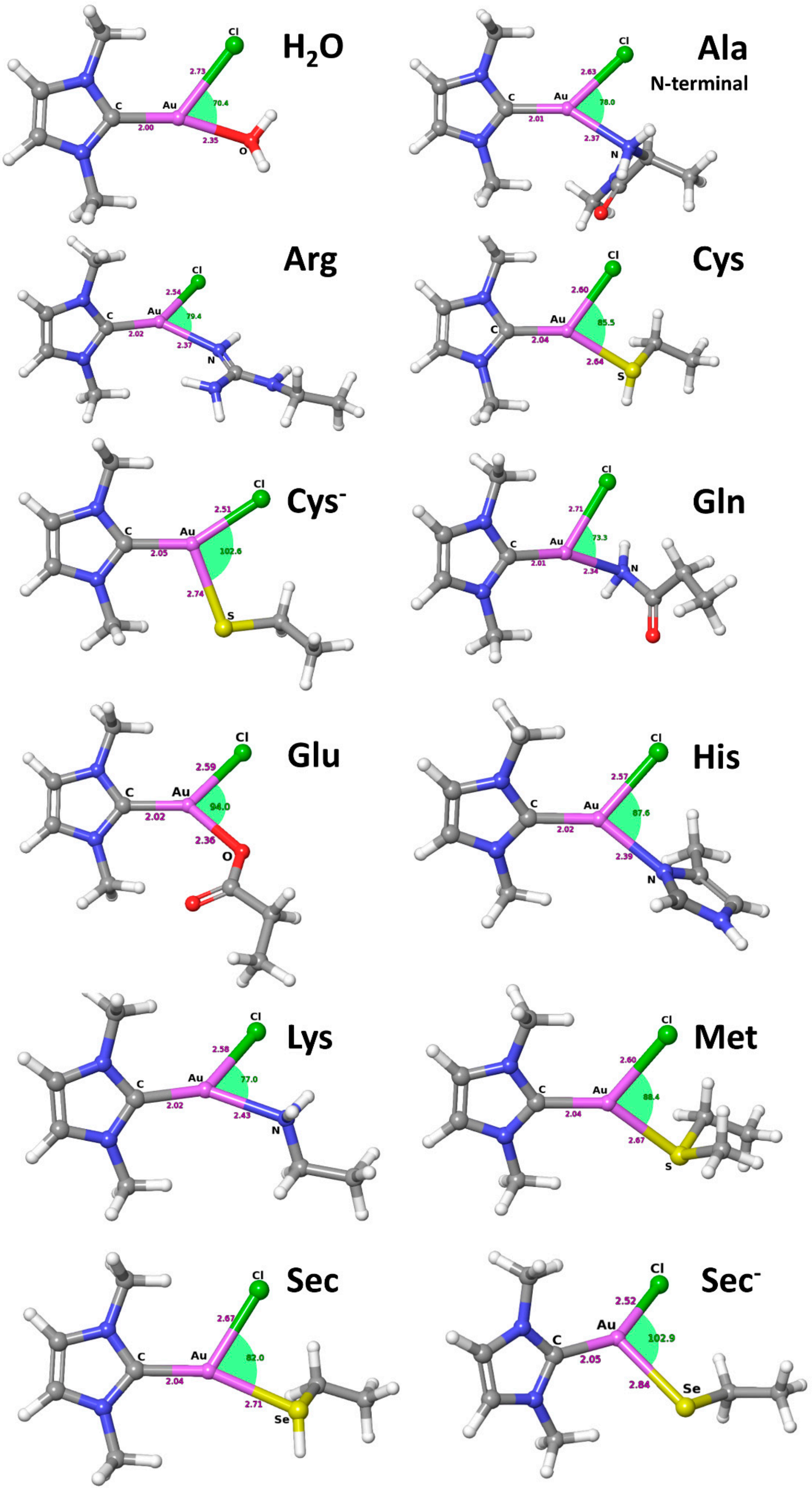

Figure S2. Transition states for various nucleophiles. All distances in angstroms, all angles in degrees.

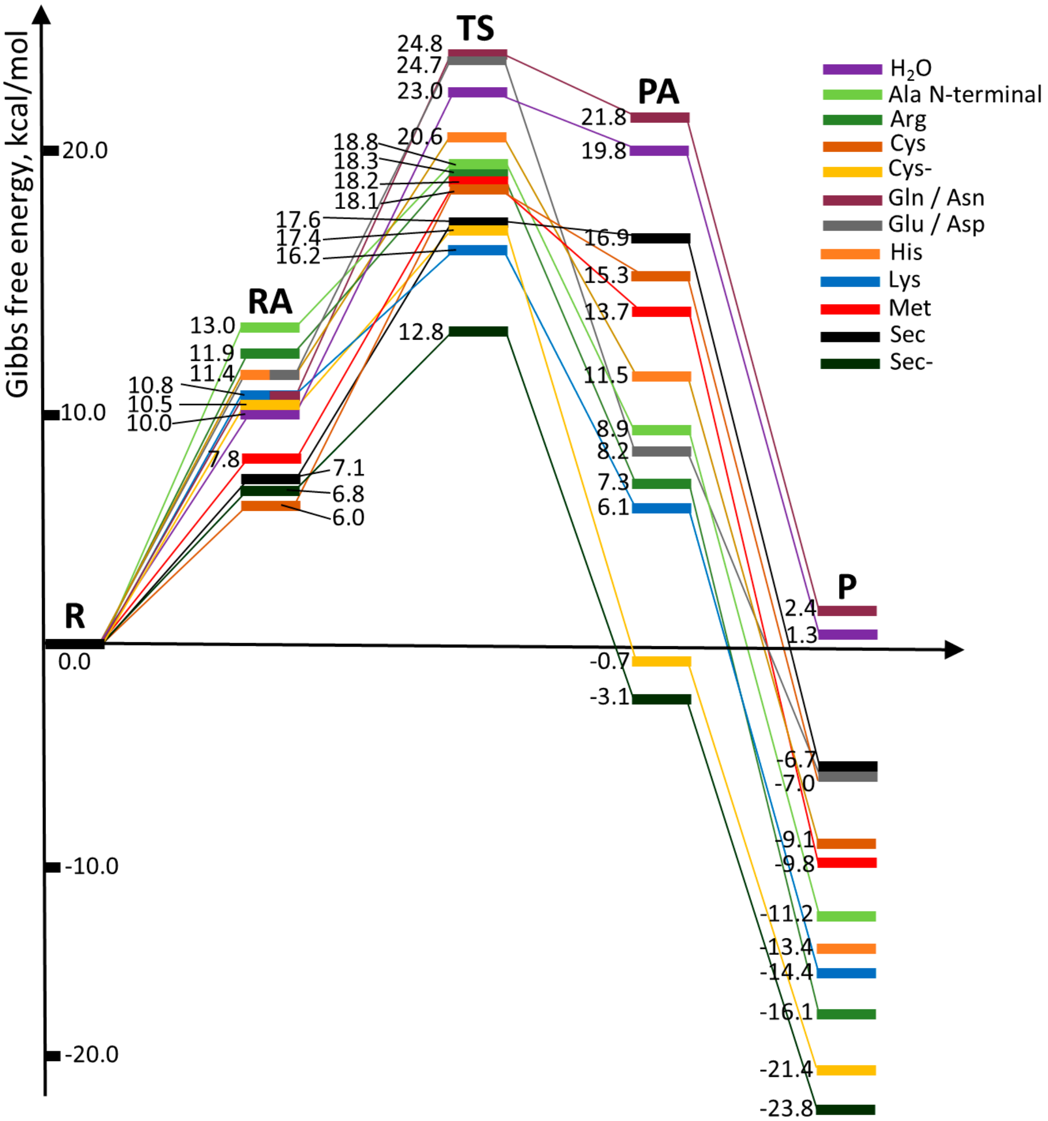

Figure S3. Reaction profiles for IMeAuCl interacting with various nucleophiles. Values in kcal/mol are calculated at the B3LYP/LACV3P**++//B3LYP/LACVP** level. R, P, TS, RA, and PA stand for reactant, product, transition state, reactant-adduct, and product-adduct, respectively.

Transition States for:

Dissociation of Cl

Au	-0.014904	0.002348	0.011039
Cl	3.724822	0.047590	0.010883
C	-2.018369	-0.022896	0.011196
N	-2.822041	-1.112841	-0.049385
C	-4.153142	-0.728909	-0.028317
C	-4.171158	0.626415	0.047864
N	-2.850798	1.045413	0.072154

H	-4.958343	-1.445286	-0.068999
H	-4.995037	1.321348	0.086860
C	-2.372074	-2.502888	-0.128262
H	-2.749109	-3.063077	0.730776
H	-2.734000	-2.957441	-1.053562
H	-1.282744	-2.519053	-0.119929
C	-2.438651	2.446956	0.153857
H	-2.827019	2.997235	-0.706376
H	-2.816204	2.891267	1.077913
H	-1.350196	2.492171	0.149973

H_2O

Au	0.042778	0.139011	2.322318
C	-4.096982	0.258619	1.688258
C	-3.999555	1.353552	2.484588
N	-2.662293	1.481810	2.824207
C	-1.924691	0.491538	2.252979
N	-2.819329	-0.257193	1.553772
H	-4.751629	2.043675	2.832832
Cl	1.429172	-1.673958	0.830343
H	-4.952293	-0.193782	1.211906
C	-2.129677	2.549225	3.663176
H	-2.664315	2.575224	4.616295
H	-1.074355	2.343123	3.840824
H	-2.233977	3.514509	3.159618
C	-2.498303	-1.459633	0.780031
H	-2.920728	-1.365845	-0.223260
H	-1.413834	-1.564916	0.711337
H	-2.918021	-2.340442	1.273387
O	2.319329	0.211462	2.905190
H	2.457041	-0.563190	2.298311
H	2.696454	0.960963	2.422905

Alanine N-terminal

Au	0.0683062008	0.0599121847	2.1900937165
C	-4.1202480450	0.3703300155	1.7902220698
C	-3.9144553361	1.4342416631	2.6084713703
N	-2.5515170090	1.4929304076	2.8530034874
C	-1.8987943702	0.4882791862	2.2040473367
N	-2.8795157037	-0.1991723246	1.5556738861

H	-4.6086853166	2.1409990012	3.0357470135
Cl	1.4974357093	-2.0841163137	1.6733697855
H	-5.0282346768	-0.0294604346	1.3665504450
C	-1.9119315306	2.5197010013	3.6718509976
H	-2.4256868895	2.5948139079	4.6342499597
H	-0.8695138983	2.2463467187	3.8357188139
H	-1.9547123947	3.4877868370	3.1636470865
C	-2.6568831260	-1.3770193583	0.7190804637
H	-2.8167983091	-1.1278951583	-0.3339228871
H	-1.6290190647	-1.7148007363	0.8616068607
H	-3.3479235624	-2.1707920522	1.0141554069
C	1.9686190702	0.9684690613	4.9879328838
C	2.7952867372	0.3188599737	3.8726041376
N	2.2768338964	0.8301232335	2.5929971925
C	4.2896688996	0.6155101459	4.0854367549
H	2.6306550810	-0.7635974089	3.8475532982
H	2.3359859346	1.8460730670	2.5802755192
H	2.7993788494	0.4275609773	1.8184684879
H	4.8806879305	0.1661944176	3.2816551354
H	4.4727963147	1.6949297119	4.0976366716
H	4.6412449694	0.1995100723	5.0361245329
N	1.6968160051	0.1565104541	6.0450643911
O	1.6295871279	2.1505980307	4.9285120513
C	0.9598169878	0.6047828466	7.2137518421
H	1.9164271578	-0.8249677388	5.9566188351
H	1.5336236300	0.4353404252	8.1321024611
H	0.7824310212	1.6743423057	7.0954983928
H	-0.0034380799	0.0888236364	7.3002611512

Arginine

Au	-0.2098533167	-0.1312548723	2.2164225906
C	-4.0425179985	0.5762535440	0.5821909913
C	-4.4014857461	0.4540070801	1.8838836809
N	-3.2343421024	0.2251076364	2.5991435370
C	-2.1491519602	0.1928786226	1.7708173792
N	-2.6670725643	0.4098324159	0.5279794977
H	-5.3692666370	0.5019113154	2.3576337536
Cl	1.7688442121	-1.5729103150	1.5346846745
H	-4.6362377238	0.7616579669	-0.2988456178
C	-3.1893056426	0.0007885772	4.0373538036
H	-3.6466629351	-0.9600838249	4.2919019525

H	-2.1438435208	-0.0103778986	4.3463787809
H	-3.7216759591	0.8027931003	4.5575826777
C	-1.8761581752	0.4689939799	-0.6973383229
H	-1.6728859406	1.5073843810	-0.9763239334
H	-0.9322583595	-0.0494421072	-0.5235788386
H	-2.4231790243	-0.0243033005	-1.5044666693
N	2.7736945587	2.1302109385	5.0521974739
C	1.6384937244	1.8113772650	4.3288085516
N	1.4703643278	0.7202667446	3.6605090443
N	0.6329687863	2.7644048683	4.3572445144
H	2.2945885973	0.1397243593	3.5211669488
H	3.5264210997	1.4703243476	4.9033394111
C	3.2202158097	3.5132008006	5.2529091064
H	-0.2121211955	2.4221541503	3.9161906306
H	0.4768489451	3.2160042719	5.2486056599
C	4.5618310071	3.5267198349	5.9799197230
H	3.2975164689	4.0508526434	4.2969689842
H	2.4792344275	4.0481513467	5.8582889329
H	4.8899192623	4.5548629245	6.1555655474
H	4.4884114357	3.0175583806	6.9455945782
H	5.3374178170	3.0280181589	5.3874663776

Cysteine

Au	-0.1787508576	0.1085659420	2.3017229792
C	-4.0038510136	-0.4539075367	0.5885199713
C	-4.4081368706	0.1427456419	1.7388191715
N	-3.2624685137	0.4061696827	2.4729294382
C	-2.1500950927	-0.0123255005	1.8065312722
N	-2.6227229110	-0.5389345382	0.6434570011
H	-5.3936600429	0.4031836553	2.0917705846
Cl	1.8235668574	-0.3315698333	0.6984858337
H	-4.5704810090	-0.8209434111	-0.2528376538
C	-3.2600951522	1.0611976804	3.7743119439
H	-3.9337848775	0.5373334570	4.4577754513
H	-2.2423219355	1.0293596006	4.1620150344
H	-3.5798457977	2.1023989377	3.6776677514
C	-1.7976817633	-1.1331219228	-0.4110803992
H	-2.1085435077	-0.7309881624	-1.3786096466
H	-0.7490346671	-0.8876158527	-0.2288356983
H	-1.9207389130	-2.2201684374	-0.4129889216
C	3.9467283851	-0.8174237346	4.9993359637

C	2.7742775358	-0.8928173415	4.0283836711
S	1.6596441059	0.5801464135	4.1328038584
H	4.5462007967	0.0805761458	4.8267610092
H	3.0831888555	-0.8863882845	2.9801426945
H	2.1646419007	-1.7855555496	4.1828227214
H	1.0617235510	0.2630224092	5.3013346049
H	3.6150284672	-0.8108612039	6.0432540850
H	4.5937434982	-1.6911793169	4.8600428765

Glutamic Acid

Au	-0.099885	-0.222925	2.281731
C	-3.941199	0.677610	0.734397
C	-4.111801	1.160510	1.991952
N	-2.921774	0.950911	2.669387
C	-2.000666	0.337922	1.871143
N	-2.651399	0.173281	0.677346
H	-4.963937	1.630858	2.457789
Cl	1.436988	-2.046044	1.278953
H	-4.611433	0.654095	-0.110869
C	-2.689792	1.305293	4.068821
H	-2.991247	0.477565	4.719984
H	-1.623208	1.531509	4.221619
H	-3.293707	2.186127	4.307893
C	-2.052279	-0.442133	-0.499396
H	-1.794927	0.318853	-1.244017
H	-1.143480	-0.961634	-0.184951
H	-2.757321	-1.154080	-0.939991
C	2.504797	2.075529	5.347528
C	1.288734	1.573974	4.524448
O	1.566860	0.619284	3.729592
O	0.190637	2.144539	4.715030
H	3.257035	2.435509	4.633499
C	2.165515	3.160628	6.369845
H	1.705746	4.023044	5.878005
H	1.437405	2.792539	7.100092
H	2.958678	1.203365	5.833980
H	3.057871	3.501368	6.913010

Glutamine

Au	-0.228586	0.098253	2.412592

C	-3.931282	-0.344101	0.470222
C	-4.218372	0.782450	1.171690
N	-3.083797	1.098026	1.901157
C	-2.096401	0.190381	1.670132
N	-2.629592	-0.694292	0.785230
H	-5.115995	1.378839	1.214924
Cl	1.610078	-1.809041	1.832366
H	-4.531111	-0.920335	-0.216571
C	-2.977681	2.248430	2.791910
H	-3.723967	2.177411	3.587663
H	-1.977988	2.248949	3.225889
H	-3.131678	3.173127	2.229542
C	-1.938218	-1.860471	0.228505
H	-1.914283	-1.783777	-0.861560
H	-0.917825	-1.894163	0.615529
H	-2.466573	-2.772381	0.518601
C	3.196994	1.743653	2.112842
C	2.283078	1.851374	3.321655
N	1.685355	0.629784	3.719503
O	2.048103	2.881945	3.921399
H	2.150111	-0.238581	3.425311
H	2.816154	0.967985	1.438631
C	3.397659	3.084217	1.406397
H	2.459675	3.441806	0.970109
H	3.746254	3.845705	2.108470
H	1.388416	0.644166	4.690760
H	4.158869	1.360516	2.484151
H	4.131282	2.985809	0.601206

Histidine

Au	-0.1782903518	-0.0538610309	2.1241990553
C	-4.1455486783	0.2819852259	0.7298998648
C	-4.3833276206	0.5751243420	2.0333319332
N	-3.1570157104	0.5354866119	2.6795425022
C	-2.1560029106	0.2170335372	1.8099030752
N	-2.7832453969	0.0608992232	0.6102435074
H	-5.3026503688	0.8004178265	2.5505173593
Cl	1.5426743083	-1.4901718661	0.8680104719
H	-4.8174450236	0.2122001331	-0.1109762113
C	-2.9727049993	0.7711149034	4.1046571520
H	-3.3668440148	-0.0665582457	4.6873368156

H	-1.9031258851	0.8690856493	4.2925885307
H	-3.4869089714	1.6901904726	4.3985651893
C	-2.1069252188	-0.2787866889	-0.6417971874
H	-1.9525954765	0.6197646310	-1.2473433074
H	-1.1392241687	-0.7270493549	-0.4076953920
H	-2.7202091956	-0.9917614530	-1.1984410384
N	1.6310838007	0.7750854877	3.4533502338
C	2.5785113212	0.0706133004	4.1776046396
C	3.5625104096	0.9414591634	4.5730871680
N	3.2035376996	2.1822128247	4.0764601631
C	2.0326541878	2.0304875298	3.4077144408
H	1.5220593021	2.8393145680	2.9066004930
H	3.7264953466	3.0386664121	4.1722956402
H	4.4653789905	0.7915706333	5.1440139453
C	2.4609016372	-1.3989433746	4.4125017061
H	3.3203143677	-1.7712248848	4.9776565210
H	1.5498683295	-1.6344549890	4.9754930262
H	2.4012528027	-1.9204735573	3.4511267383

Lysine

Au	-0.1550946211	-0.0805414437	2.1661259303
C	-4.0675438633	0.5090818921	0.7041006388
C	-4.3536562003	0.5219515952	2.0298958637
N	-3.1494018850	0.3595274911	2.6988193347
C	-2.1146919855	0.2365747805	1.8194702181
N	-2.6981112978	0.3286186829	0.5909810162
H	-5.2932572767	0.6254888617	2.5492488388
Cl	1.7429338631	-1.5212185478	1.1879421301
H	-4.7086585395	0.6099676642	-0.1572704135
C	-3.0217083571	0.2864835212	4.1478246011
H	-3.3828918148	-0.6771578972	4.5192583772
H	-1.9669524750	0.3957641150	4.3999855585
H	-3.5969197336	1.0928324653	4.6102847654
C	-1.9787094563	0.2617532982	-0.6787967146
H	-1.8123084716	1.2663011838	-1.0794042777
H	-1.0171854632	-0.2244967276	-0.5083786221
H	-2.5629863378	-0.3227169351	-1.3936700656
C	3.1790407836	0.9751496147	5.5136039798
C	1.8155504946	0.6375628257	4.9042853321
N	1.7880452480	0.6441031864	3.4323958577
H	1.4925164519	-0.3569934546	5.2297280555

H	1.0593986251	1.3479043638	5.2582437539
H	2.0889211056	1.5453506299	3.0674799157
H	2.4034287793	-0.0664544906	3.0286099458
H	3.5064356213	1.9790959870	5.2185666470
H	3.1362116413	0.9462001785	6.6083423969
H	3.9429825854	0.2616548293	5.1868143737

Methionine

Au	-0.1425441801	0.1069984109	2.1692002469
C	-4.1036732638	-0.3710111034	0.7439390166
C	-4.4072943437	0.0382489565	2.0018171282
N	-3.2019512242	0.2597691938	2.6492622184
C	-2.1501491791	-0.0038947150	1.8244217613
N	-2.7219667720	-0.3924436536	0.6514712661
H	-5.3597123542	0.1875513908	2.4857171084
Cl	1.7989275027	-0.5510568397	0.5763874489
H	-4.7411212720	-0.6438055095	-0.0822344002
C	-3.0876213101	0.7061802483	4.0322483232
H	-3.4982868075	-0.0484015528	4.7092642207
H	-2.0300412085	0.8544117493	4.2502073420
H	-3.6268677676	1.6478489788	4.1679223291
C	-1.9901803717	-0.7729678666	-0.5576452180
H	-2.1990861792	-0.0563033926	-1.3565281609
H	-0.9199774249	-0.7735012530	-0.3399282743
H	-2.3011709481	-1.7723219474	-0.8737582034
C	3.9384047682	-0.6274184471	4.9119439171
C	2.6783251636	-0.7384388847	4.0567456921
S	1.6864972155	0.8130423062	3.9840093223
H	1.9846636079	-1.4753562495	4.4725736444
C	2.7767014323	1.8889504419	2.9839711903
H	2.2407157417	2.8296334649	2.8442031901
H	3.7097052257	2.0839190590	3.5160710375
H	2.9507387110	1.4150473905	2.0154902799
H	2.8853777712	-1.0254116602	3.0217002907
H	4.4509957209	-1.5955880793	4.9367818300
H	3.7043109429	-0.3383700932	5.9410272647
H	4.6434791653	0.1025041112	4.5042911988

Selenocysteine

Au	-0.182328	0.148844	2.390135

C	-3.862560	-0.647062	0.454023
C	-4.339475	-0.526681	1.718954
N	-3.249885	-0.248941	2.528558
C	-2.102116	-0.194522	1.796369
N	-2.494893	-0.442542	0.517889
H	-5.341678	-0.612719	2.108211
Cl	1.848635	0.040880	0.661616
H	-4.369863	-0.856456	-0.474460
C	-3.336037	-0.050461	3.969610
H	-3.686223	-0.964943	4.457311
H	-2.338454	0.198457	4.331843
H	-4.025070	0.768502	4.195453
C	-1.611482	-0.484032	-0.650536
H	-1.889630	0.310871	-1.347976
H	-0.576341	-0.342060	-0.329630
H	-1.711025	-1.453744	-1.145044
C	4.213560	-0.857126	4.723438
C	2.951164	-0.886130	3.878136
Se	1.813006	0.772762	4.114309
H	4.831737	0.014022	4.489701
H	3.136365	-0.868645	2.802081
H	2.304298	-1.734507	4.106833
H	1.294524	0.392058	5.451015
H	3.994446	-0.845693	5.796225
H	4.805328	-1.756641	4.514638

Selenolate

Au	0.177958	-0.244728	2.019334
C	-3.917562	0.513564	1.024715
C	-4.042674	0.292375	2.357742
N	-2.777227	-0.010542	2.835852
C	-1.848089	0.000926	1.831688
N	-2.576795	0.328167	0.718349
H	-4.909170	0.326553	3.000097
Cl	2.250092	-1.342118	1.103151
H	-4.651661	0.787845	0.282926
C	-2.473743	-0.337170	4.225683
H	-2.635079	-1.404649	4.412379
H	-1.424846	-0.087896	4.428092
H	-3.127734	0.247515	4.879347
C	-2.003229	0.466903	-0.610081

H	-1.910099	1.522153	-0.888677
H	-1.009847	0.013758	-0.587586
H	-2.634813	-0.045958	-1.343080
C	4.037529	1.041595	4.933217
C	2.887625	0.091552	4.612044
Se	1.133059	1.064561	4.345563
H	4.184106	1.757283	4.117546
H	3.078272	-0.459649	3.689212
H	2.749155	-0.630435	5.423401
H	3.838396	1.618369	5.844872
H	4.981648	0.492149	5.078895

Thiolate

Au	-0.001452	-0.135580	1.894538
C	-4.213869	0.114031	1.151142
C	-4.211546	0.313765	2.494487
N	-2.889221	0.266636	2.905216
C	-2.046869	0.043916	1.853669
N	-2.889159	-0.047818	0.775957
H	-5.024735	0.481226	3.183729
Cl	2.090664	-0.561888	0.580413
H	-5.029300	0.068724	0.445511
C	-2.442119	0.468209	4.282174
H	-3.266496	0.216834	4.955961
H	-1.568518	-0.159781	4.482195
H	-2.145199	1.509036	4.436898
C	-2.440234	-0.290916	-0.586678
H	-2.727071	0.543064	-1.236283
H	-1.351226	-0.380344	-0.557436
H	-2.879033	-1.217499	-0.972380
C	3.673505	0.570068	4.996609
C	2.689440	-0.438884	4.399526
S	0.943776	0.155205	4.447284
H	3.637195	1.511070	4.437650
H	2.951402	-0.642436	3.356837
H	2.760758	-1.386736	4.949868
H	3.422608	0.797441	6.039880
H	4.707624	0.192220	4.968187

© 2019 by the authors. Licensee MDPI, Basel, Switzerland. This article is an open access article distributed under the terms and conditions of the Creative Commons Attribution (CC BY) license (http://creativecommons.org/licenses/by/4.0/).
